# Facile Synthesis of Pyrazole- and Benzotriazole-Containing Selenoethers

**DOI:** 10.1155/2014/578762

**Published:** 2014-11-20

**Authors:** Andrei S. Potapov, Nina P. Chernova, Vladimir D. Ogorodnikov, Tatiana V. Petrenko, Andrei I. Khlebnikov

**Affiliations:** ^1^Department of Biotechnology and Organic Chemistry, National Research Tomsk Polytechnic University, 30 Lenin Avenue, Tomsk 634050, Russia; ^2^Department of Chemistry, Altai State Technical University, 46 Lenin Avenue, Barnaul 656038, Russia; ^3^Institute of Petroleum Chemistry, Siberian Branch of Russian Academy of Sciences, 3 Akademicheskii Avenue, Tomsk 634055, Russia

## Abstract

Azole-containing selenoethers, 1,5-bis(3,5-dimethylpyrazol-1-yl)-3-selena pentane and 1,3-bis(1,2,3-benzotriazol-1-yl)-2-selena propane were prepared by the reaction of corresponding tosylate or chloride with sodium selenide generated *in situ* from elemental selenium and sodium formaldehydesulfoxylate (rongalite).

## 1. Introduction

Organoselenium compounds find applications due to their biological activity and useful synthetic properties (see [1, 2] and references cited therein). Selenoethers demonstrate potent ligating ability towards transition and main-group elements [3]. On the other hand, azole-containing thioethers are also known for their rich coordination chemistry [4]. Therefore, ligands carrying both azole- and selenoether moieties are especially interesting in view of their coordination chemistry. Nevertheless, only a few reports on compounds of this type have appeared in literature, demonstrating their use as building blocks for supramolecular architecture [5] and as ligands for catalysts [6–9]. Hodage et al. demonstrated potential glutathione peroxidase-like activity of some pyrazole-containing selenoethers [10]. Recently, Pop et al. prepared a series of late transition metal complexes of pyrazole-derived selenoethers [11].

Dialkyl selenides (selenoethers) are usually prepared from alkyl halides and Se^2−^ species, generated from various selenium compounds. Since selenide ions are very unstable towards oxygen, they are generated* in situ* using different reducing agents. Selenium in combination with aqueous NaOH [12], liquid ammonia and sodium [13], sodium in DMF [14], and sodium formaldehydesulfoxylate (rongalite) [15] were reported as sources of selenide ions. Other selenium compounds, such as selenium dioxide (reduced by trialkyl borohydrides) [16] or selenium tetrachloride [17], are less commonly used.

Herein we report improved methods for the preparation of pyrazole- and benzotriazole-containing selenoethers 1,5-bis(3,5-dimethylpyrazol-1-yl)-3-selena pentane (**2**) and 1,3-bis(1,2,3-benzotriazol-1-yl)-2-selena propane (**4**).

## 2. Results and Discussion

### 2.1. Synthesis of Selenoethers

**Scheme 1:**
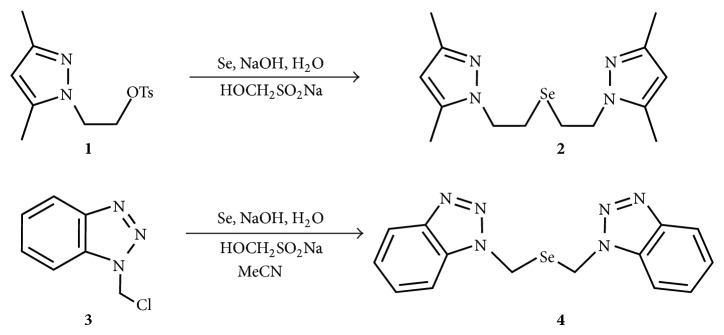
Synthesis of azole-selenoethers.

In our preparation of azole-containing selenoethers we used elemental selenium and sodium formaldehydesulfoxylate (HOCH_2_SO_2_Na, rongalite) in aqueous NaOH [18]. The generated* in situ* sodium selenide was introduced into the reaction with 1-(2-tosyloxy ethyl)-3,5-dimethylpyrazole (**1**) or 1-chloromethyl benzotriazole (**3**) (Scheme 1). Due to low solubility of compound** 3** in water acetonitrile was added to the reaction mixture in order to expedite the nucleophilic substitution. It should be noted that we found it unnecessary to carry out the reactions under nitrogen atmosphere, which is probably due to reductive atmosphere created by SO_2_ evolution from the excess of rongalite. Pyrazole- and benzotriazole-containing selenoethers (**2** and** 4**) were obtained in good yields (76 and 90%) as off-white air- and moisture-stable solids even in the absence of nitrogen atmosphere. It should be noted that in our synthetic procedure selenide ions were generated using inexpensive and stable rongalite in contrast to superhydride (LiBEt_3_H) or NaBH_4_ used in previously reported methods of preparation of selenoethers** 2** [10] and** 4** [19]. The structures of selenoethers were confirmed by IR and NMR spectroscopy and, in case of selenoether** 2**, electron-impact mass-spectrometry.

It is known [20] that upon reduction selenium can form diselenide ions Se_2_^2−^ in addition to selenides Se^2−^. Therefore, not only selenoethers, but also diselenides can form as a result of reactions in Scheme 1, and IR and NMR spectroscopy alone do not allow to unambiguously discern between them.

### 2.2. X-Ray Crystal Structure Determination

In order to establish the structures of compounds** 2** and** 4** we have carried out single crystal X-ray structure determinations. Single crystals of compound** 4** were obtained by crystallization from acetonitrile. Compound** 2** has a relatively low melting point and crystallized rapidly from various solvents, preventing the formation of single crystals. However, with copper(II) nitrate compound** 2** readily gave well-formed crystals of complex suitable for X-ray structure determination. The complex [Cu(**2**)(NO_3_)_2_] (**5**) was obtained in high yield (84%); therefore selenoether** 2** and not some other impurity acted as a ligand and the structure of the complex can be used for the elucidation of compound** 2** structure.

**Figure 1:**
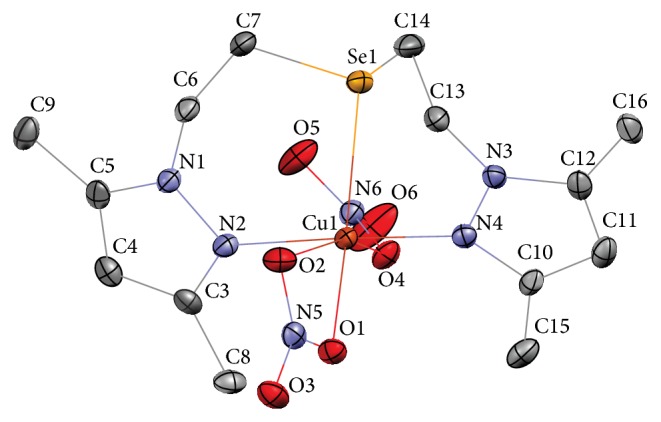
Molecular structure of compound** 5**. Thermal ellipsoids for nonhydrogen atoms are drawn at 50% probability level. Hydrogen atoms are omitted for clarity.

Complex** 5** crystallizes in a monoclinic crystal system; crystallographic parameters and details of the diffraction experiment are given in Table 1. Molecular structure of the complex is shown in Figure 1, and selected bond lengths and angles are listed in Table 2. From the structure of complex** 5** it is evident that compound** 2** is indeed a selenoether and not a diselenide. The lengths of C–C and C–N bonds in pyrazole rings are within the usual range [21]. The lengths of Se–C bonds (1.95-1.96 Å) are also common for acyclic selenoethers [22].

Reports on the synthesis and crystal structure of benzotriazole-containing selenoether** 4** have appeared in two recent papers. Lu et al. [23] used a nucleophilic substitution reaction of pure sodium selenide with chloro-derivative** 2** to prepare the selenoether in 55% yield. Das et al. [19] improved the yield up to 78% by generating Na_2_Se* in situ* from selenium and sodium borohydride. Both papers report crystal structures of prepared selenoethers, which they describe as pale-yellow crystals (m.p. 140°C [19]), readily soluble in common organic solvents. Both products appear to be the same monoselenide, and the slight differences in crystal structures are probably due to unlike packing fashion of formula units in elementary cells (monoclinic crystal system).

**Figure 2:**
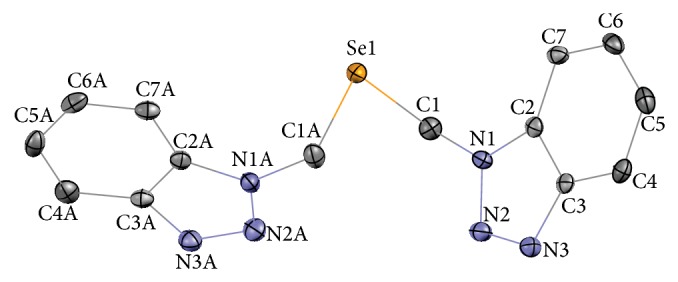
Molecular structure of selenoether** 4**. Thermal ellipsoids for nonhydrogen atoms are drawn at 50% probability level. Hydrogen atoms are omitted for clarity.

**Figure 3:**
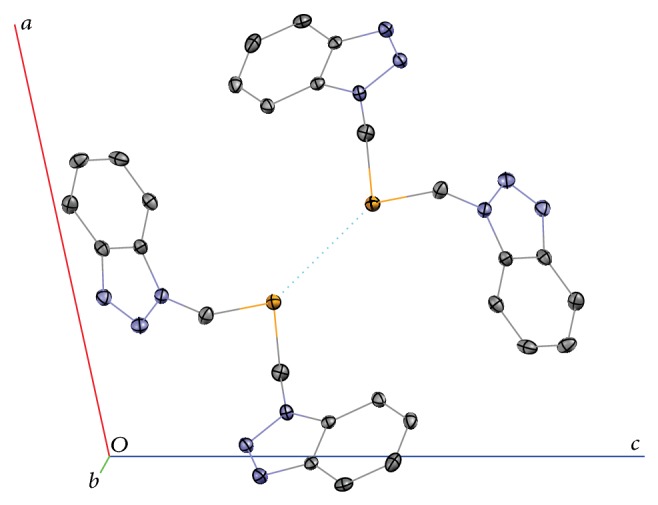
Se–Se intermolecular contacts in the structure of** 4**. Some molecules in the unit cell are not shown for clarity.

The crystallographic parameters, bond lengths, and angles for compound** 4** are given in Tables 1 and 2. The asymmetric unit of this compound is a monoselenide (Figure 2), and the elementary cell contains four such units. The molecular structure of selenoether** 4** is very similar to those reported by Das et al. and Lu et al. [19, 23]. The lengths of Se–C bonds are slightly (by 0.01 Å) longer than in previously reported structures, while C–Se–C angle is slightly sharper. The major type of intermolecular interactions, that is, probably responsible for low solubility and high melting point of compound** 4**, is Se–Se contacts (3.7936(3) Å, Figure 3), the length of which is in the range reported previously for selenoethers [24].

## 3. Conclusion

In summary, two selenoethers (pyrazole- and benzotriazole-containing,** 2** and** 4**) were prepared using elemental selenium-rongalite system for* in situ* selenide ion generation. The proposed method uses inexpensive reagents, and provides higher yields compared to reported procedures.

## 4. Experimental

Elemental analyses were carried out on a Carlo Erba analyzer. Infrared (IR) spectra of solid samples as KBr pellets were recorded on a Nicolet 5700 (4000–400 cm^−1^) spectrophotometer. NMR spectra were recorded on Bruker AV300 instrument operating at 300 MHz for ^1^H and 75 MHz for ^13^C. EI MS measurements were carried out using TRACE DSQ (Thermo Electron Corporation, USA) instrument.

Single crystals of compounds** 4** and** 5** for crystal structure determination were mounted in inert oil and transferred to the cold gas stream of the diffractometer. The structure was determined at 153 K by conventional single crystal X-ray diffraction techniques using an automated four-circle Bruker-Nonius X8 Apex diffractometer equipped with a 2D CCD detector and graphite monochromated molybdenum source (*λ* = 0.71073 Å). Intensity data were collected by *φ*-scanning of narrow frames (0.5°) to 2*θ* = 54.96°. Absorption correction was applied empirically by the program SADABS [25]. The structure was solved by the direct method and refined using the full-matrix least-squares technique in the anisotropic approximation for nonhydrogen atoms with the program package SHELX-97 [26]. Hydrogen atoms were localized geometrically.

Tosylate** 1** [27] and chloro-derivative** 3** [28] were prepared according to known procedures; sodium formaldehydesulfoxylate dihydrate (rongalite) was purchased from Acros.

### 4.1. 1,5-Bis(3,5-dimethylpyrazol-1-yl)-3-selena Pentane (2)

A suspension of selenium (0.395 g, 5 mmol), sodium formaldehydesulfoxylate dihydrate (3.08 g, 20 mmol), and NaOH (1.10 g, 27.5 mmol) in water (5 mL) was stirred at room temperature, until the initially formed red solution turned colorless and white precipitate of Na_2_Se was formed (15–20 min). Tosylate** 1** (2.94 g, 10 mmol) was then added in one portion, the mixture was brought to reflux and stirring was continued for 3 hours (TLC control). After that water (30 mL) was added to the reaction mixture to dissolve the precipitated product and excess of rongalite. The solution obtained was extracted with chloroform (5 × 20 mL); the extract was dried over anhydrous Na_2_SO_4_. After removal of solvent, slightly yellow oil was obtained, which crystallized on standing at room temperature. The product was recrystallized form hexane to give colorless crystals of selenoether** 2**. Yield 1.24 g (76%), mp 54–56°C (hexane). IR (*ν*, cm^−1^) 1550, 1460 (*ν*_Pz_), 1298 (*δ*_C–H_, Pz), 1026 (Pz breating), 776 (*ν*_C–Se_). ^1^H NMR (CDCl_3_): 2.16 (s, 6H, 3-CH_3_-Pz), 2.21 (s, 6H, 5-CH_3_-Pz), 2.81 (t, 4H, *J* = 7 Hz, PzCH_2_CH_2_Se), 4.10 (t, 4H, *J* = 7 Hz, PzCH_2_CH_2_Se), 5.73 (s, 2H, 4-H-Pz). ^13^C NMR (CDCl_3_): 11.0 (5-CH_3_-Pz), 13.3 (3-CH_3_-Pz), 23.3 (PzCH_2_ CH_2_Se), 48.9 (PzCH_2_CH_2_Se), 104.8 (4-C-Pz), 138.8 (5-C-Pz), 147.5 (3-C-Pz). EI-MS (70 eV): 326 (M^+^), 230 ([M-Pz]^+^), 203 ([M-PzCH_2_CH_2_]^+^), 109 ([PzCH_2_]^+^). Anal. Calc'd for C_14_H_22_N_4_Se (325.31): C, 51.59; H, 6.82; N, 17.22. Found: C, 51.97; H, 7.01; N, 17.70.

### 4.2. 1,3-Bis(1,2,3-benzotriazol-1-yl)-2-selena Propane (4)

Selenoether** 4** was prepared similarly to compound** 2** from 2.10 g (12.54 mmol) chloro-derivative** 1**, 0.50 g (6.27 mmol) of selenium, 2.31 g (15.0 mmol) of sodium formaldehydesulfoxylate dihydrate, and 1.38 g (34.5 mmol) of NaOH in 6 mL of water and 15 mL of acetonitrile. Yield 1.94 g (90%), colorless crystals, mp 182-183°C (DMF). IR (*ν*, cm^−1^) 1612, 1496, 1453 (*ν*_Bta_), 754 (*ν*_C–Se_). ^1^H NMR (DMSO-d_6_): 6.19 (s, 4H, CH_2_), 7.44 (t, 2H, 5-H-Bta, *J* = 7.5 Hz), 7.58 (t, 2H, 6-H-Bta, *J* = 7.5 Hz), 7.97 (d, 2H, 4-H-Bta, *J* = 8 Hz), 8.08 (d, 2H, 7-H-Bta, *J* = 8 Hz). ^13^C NMR (DMSO-d_6_): 42.5 (CH_2_), 111.1 (7-C-Bta), 119.3 (4-C-Bta), 124.4 (5-C-Bta), 127.5 (6-C-Bta), 131.9 (8-C-Bta), 145.4 (9-C-Bta). Anal. Calc'd for C_14_H_12_N_6_Se (343.25): C, 48.99; H, 3.52. Found: C, 49.30; H, 3.83.

### 4.3. 1,5-Bis(3,5-dimethylpyrazol-1-yl)-3-selena Pentane-Dinitrato Copper (5)

To a solution of selenoether** 2** (0.065 g, 0.2 mmol) in acetone (0.2 mL), solution of Cu (NO_3_)_2_·3H_2_O (0.048 g, 0.2 mmol) in acetone (0.2 mL) was added. After standing for 2 hours, deep-green crystals of the complex were formed, which were filtered, washed with acetone, and dried. The crystals were suitable for X-ray crystal structure determination. Yield 0.086 g (84%). IR (*ν*, cm^−1^) 1556 (*ν*_Pz_), 1026 (Pz breating), 811 (*ν*_C–Se_). Anal. Calc'd for C_14_H_22_CuN_6_O_6_Se (512.87): C, 32.79; H, 4.32; N, 16.39. Found: C, 33.04; H, 4.50; N, 15.96.

## Supplementary Material

Supplementary Material contains crystallographic information files (CIF) for compounds **4** and **5**.

## Figures and Tables

**Table 1:** Crystallographic data, details of data collection, and structure refinement parameters for compounds **4** and **5**.

Example	**4**	**5**
Chemical formula	C_14_H_12_N_6_Se	C_14_H_22_CuN_6_O_6_Se
M (g mol^−1^)	343.26	512.88
Temperature (K)	100(2)	100(2)
Wavelength (Å)	0.71073	0.71073
Crystal size (mm)	0.14 × 0.11 × 0.11	0.14 × 0.11 × 0.11
Crystal system	Monoclinic	Monoclinic
Space group	P 21/c	P 21/c
*a* (Å)	11.2605(8)	16.8873(9)
*b* (Å)	9.1443(7)	8.3578(5)
*c* (Å)	13.6312(10)	13.9597(8)
*α* (°)	90	90
*β* (°)	102.2002(13)	103.2610(10)
*γ* (°)	90	90
*V* (Å^3^)	1371.90(18)	1917.74(19)
*Z*	4	4
*D*_calc_ (g cm^−3^)	1.662	1.776
*μ* (mm^−1^)	2.740	3.082
*F*(0 0 0)	688	1036
*θ* range for data collection (°)	1.85 to 29.00	2.48 to 29.00
Index ranges	−15 ≤ *h* ≤ 15	−23 ≤ *h* ≤ 23
	−12 ≤ *k* ≤ 12	−11 ≤ *k* ≤ 11
	−18 ≤ *l* ≤ 18	−19 ≤ *l* ≤ 19
Reflections collected	15771	22301
Independent reflections	3651 [*R*(int⁡) = 0.0446]	5086 [*R*(int⁡) = 0.0661]
Completeness to 2*θ* (%)	99.9	99.7
Absorption correction	Semi-empirical from equivalents	Semi-empirical from equivalents
Max. and min. transmission	0.753 and 0.700	0.913 and 0.694
Data/restraints/parameters	3651/0/190	5086/0/257
Goodness-of-fit on *F* ^2^	1.002	1.018
Final *R*_1_, *wR*_2_ [*I* > 2*σ*(*I*)]	*R*_1_ = 0.0276	*R*_1_ = 0.0351
	*wR*_2_ = 0.0575	*wR*_2_ = 0.0677
*R*_1_, *wR*_2_ (all data)	*R*_1_ = 0.0449	*R*_1_ = 0.0572
	*wR*_2_ = 0.0643	*wR*_2_ = 0.0766
Largest difference in peak and hole (e Å^−3^)	0.434 and −0.433	0.590 and −0.604

**Table 2:** Selected bond distances (Å) and angles (°) for compounds **4** and **5**.

Compound **4**			
Se(1)–C(1A)	1.960(2)	N(1A)–C(1A)–Se(1)	112.83(14)
Se(1)–C(1)	1.962(2)	C(1A)–Se(1)–C(1)	95.88(9)
N(1)–C(1)	1.440(3)	N(1)–C(1)–Se(1)	111.81(13)
N(1A)–C(1A)	1.441(3)		

Compound **5**			
Se(1)–C(7)	1.955(3)	C(7)–Se(1)–C(14)	99.87(12)
Se(1)–C(14)	1.965(3)	C(7)–Se(1)–Cu(1)	101.55(8)
Se(1)–Cu(1)	2.5110(4)	C(14)–Se(1)–Cu(1)	100.47(8)
Cu(1)–N(4)	1.965(2)	N(4)–Cu(1)–N(2)	175.68(9)
Cu(1)–N(2)	1.971(2)	N(4)–Cu(1)–O(1)	88.00(8)
Cu(1)–O(1)	2.0504(19)	N(4)–Cu(1)–Se(1)	87.11(6)
Cu(1)–O(4)	2.2700(19)	N(2)–Cu(1)–Se(1)	95.72(6)
