# Supplementary material for: Facile Synthesis of Pyrazole- and Benzotriazole-Containing Selenoethers
Source: ScientificWorldJournal. 2014 Nov 20;2014:578762. doi: 10.1155/2014/578762 (PMC4258355; doi:10.1155/2014/578762)
Supplement: Supplementary file 1 [file 578762.f1.odt]

Supporting Information

CCDC 850078 and 850079 contains the supplementary crystallographic data for this paper. These data can be obtained free of charge via

www.ccdc.cam.ac.uk/conts/retrieving.html (or from the Cambridge Crystallographic Data Centre, 12, Union Road, Cambridge CB2 1EZ, UK; fax: +44 1223 336033; or deposit@ccdc.cam.ac.uk).

Supporting Information File 1:

File Name: 850078.cif

File Format: CIF

Title: Crystal structure of compound 4

Supporting Information File 2:

File Name: 850079.CIF

File Format: CIF

Title: Crystal structure of compound 5
